# Individual Spatial Responses towards Roads: Implications for Mortality Risk

**DOI:** 10.1371/journal.pone.0043811

**Published:** 2012-09-06

**Authors:** Clara Grilo, Joana Sousa, Fernando Ascensão, Hugo Matos, Inês Leitão, Paula Pinheiro, Monica Costa, João Bernardo, Dyana Reto, Rui Lourenço, Margarida Santos-Reis, Eloy Revilla

**Affiliations:** 1 Centro de Biologia Ambiental, University of Lisboa, Lisboa, Portugal; 2 Departament of Conservation Biology, Estación Biológica de Doñana-Consejo Superior de Investigaciones Científicas, Seville, Spain; 3 Institute of Mediterranean Agrarian and Environmental Sciences, University of Évora, Évora, Portugal; The Australian National University, Australia

## Abstract

**Background:**

Understanding the ecological consequences of roads and developing ways to mitigate their negative effects has become an important goal for many conservation biologists. Most mitigation measures are based on road mortality and barrier effects data. However, studying fine-scale individual spatial responses in roaded landscapes may help develop more cohesive road planning strategies for wildlife conservation.

**Methodology/Principal Findings:**

We investigated how individuals respond in their spatial behavior toward a highway and its traffic intensity by radio-tracking two common species particularly vulnerable to road mortality (barn owl *Tyto alba* and stone marten *Martes foina*). We addressed the following questions: 1) how highways affected home-range location and size in the immediate vicinity of these structures, 2) which road-related features influenced habitat selection, 3) what was the role of different road-related features on movement properties, and 4) which characteristics were associated with crossing events and road-kills. The main findings were: 1) if there was available habitat, barn owls and stone martens may not avoid highways and may even include highways within their home-ranges; 2) both species avoided using areas near the highway when traffic was high, but tended to move toward the highway when streams were in close proximity and where verges offered suitable habitat; and 3) barn owls tended to cross above-grade highway sections while stone martens tended to avoid crossing at leveled highway sections.

**Conclusions:**

Mortality may be the main road-mediated mechanism that affects barn owl and stone marten populations. Fine-scale movements strongly indicated that a decrease in road mortality risk can be realized by reducing sources of attraction, and by increasing road permeability through measures that promote safe crossings.

## Introduction

For many species, roads represent particularly strong barriers to migration, dispersal, and genetic exchange as a result of changes in habitat quality, mortality and avoidance behavior due to traffic intensity, noise, and road surface characteristics [1], [2]. These effects entail limitations on food, shelter, and space availability, all of which are fundamental to survival and breeding performance, and may ultimately lead to reductions in population size adding to the toll of road-kills [3]. The combination of a reduction in population size and movement rates increases the probability of local extinction, and limits the capacity to adapt to future conditions [4].

To date, there has been a considerable number of studies for a wide range of species that describe patterns of road mortality and barrier effects and the environmental variables that are associated with them, thus yielding substantial insights into how some road and landscape features promote those effects [5], [6]. It is generally agreed that a higher habitat quality in the vicinity of roads may increase the probability of individuals being killed on the road [7], [8], [9]. Many road structural features are relevant, such as the availability of passages, which reduces both barrier effects and road-kills [10], and fences, which reduce the likelihood of road mortality [11]. High traffic volumes promote barrier effects [12], while road-kills are more common at intermediate intensities [13]. Nevertheless, such clear patterns offer only a partial picture, and in many cases may not help in identifying the most effective mitigation measures. For example, the factors associated with the sites at which fatalities occur may not correspond to those where animals prefer to cross [14], and studies concerning the efficacy of crossing structures to reduce barrier effects rarely evaluate whether the mitigation effort has been successful [15].

**Figure 1:**
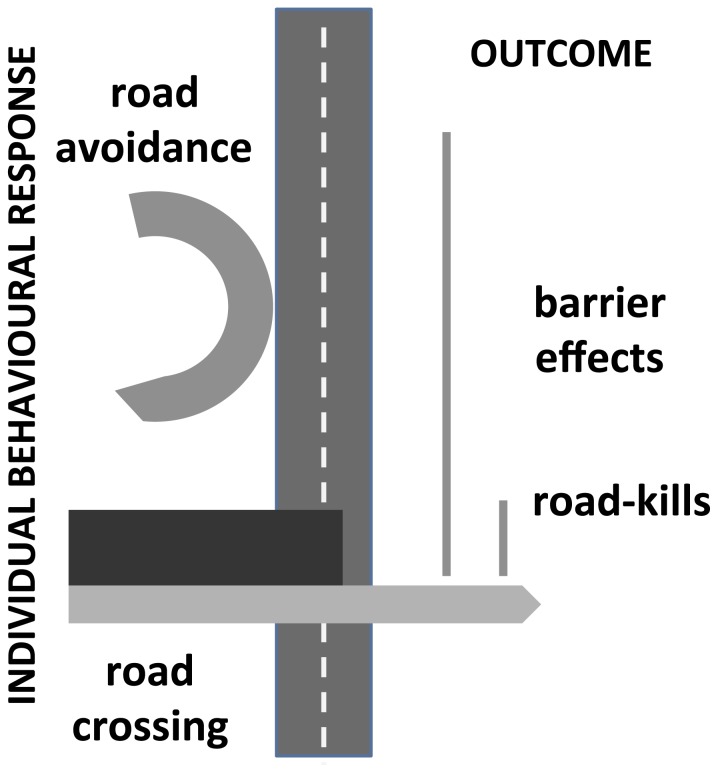
Road-kill and barrier effect.

One way to improve our ability to estimate both road-kill and barrier effects and to optimize mitigation measures is to move from the description of their patterns and covariates toward the study of individual behavioral responses to roads and the environmental variables that influence them [9], [16], [17]. Both impacts represent the outcome of a single mechanism, the individual decision either to avoid the road or to cross it (Figure 1). Studies of the behavior of individuals in the vicinity of roads would seem to provide a better understanding of the problem [18], [19], [20]. If the relative influence of road-related and landscape features on road avoidance, attraction, and the outcome of crossing (successful, unsuccessful) can be partitioned, this would seem to provide additional insight than just data on mortality, allowing the identification of the most relevant mitigation measures and their optimal spatial location. For example, COLCHERO and colleagues [21] used information derived from radio-tracking and model simulations to identify suitable locations for wildlife passes. Likewise, KLAR et al. [22] provided valuable insights for the design of fences and crossing structures using fine-scale movements to evaluate behavioral responses to traffic and crossing frequency at fenced and non-fenced sections of highways.

Some species are particularly sensitive to road-related features resulting in an almost absolute avoidance of roads (e.g., carabid beetles) [23]. In other cases, the result of the interaction between the individual and the road is conditional on factors such as traffic noise (e.g., breeding birds [24]), Thus, different behavioral responses (avoid or cross the road) are expected in species with high rates of road mortality that show some sign of spatial aggregation [25], [26]. In those cases, the identification of factors associated with road avoidance and crossing would allow for a more efficient use of mitigation measures, given that mitigating only one effect can inadvertently promote the other. Barn owls (*Tyto alba*) and stone martens (*Martes foina*) are good examples of such species with distinct behavior and ecological requirements. The barn owl is the most widely distributed owl species and is usually found in open habitats such as farmlands and grasslands associated to humanized areas [27]. Although with an acute sense of hearing to detect sound position and distance, barn owls are heavily affected by traffic [28], [29] with high rates of road mortality (49 road killed barn owls/100 km/year) [30]. In fact, in rural England barn owls have undergone a significant decline in numbers during the past century, with a 40% reduction in the total area occupied, most likely due to the presence of major roads [31]. The stone marten is a widespread species which occurs in Europe and central Asia. Although it is a forest-dwelling species associated with relatively unmodified habitats in Southern Europe [32], the stone marten is one of the carnivore species most commonly found road-killed (e.g., 8 road-killed stone martens/100 km/year on highways and two-lane paved roads [33]).

In this paper we investigate how individuals responded in their spatial behavior towards a highway and its traffic at different spatial resolutions (home range location, movement directionality and locations/crossings/road-kills). We used the barn owl and the stone marten to help identify the sources of disturbance and attraction that may not be directly evident from the patterns of road-mortality. Specifically, we assessed 1) how highways affected the species home-range location and size in the immediate vicinity of these structures, 2) which road-related features influenced their habitat selection, 3) the role of different road-related features on movement properties, and finally, 4) the characteristics associated with crossing events and road-kills. Although both species have high road mortality rates, different responses to highways may arise from their distinct use of sensory systems (auditory-visual *versus* olfactory). Barn owls may tend to avoid noisy highways whereas stone marten may ignore them. We expected habitat selection and movement properties in the vicinity of the highway to be strongly affected by traffic intensity followed by habitat suitability. Finally, suitable and continuous habitat close to the highway may explain preferences in the location of crossing events for both species. Additionally, our a priori expectation was that road features at crossing and road-kill sites should be similar if the probability of being killed was only affected by the individual crossing decisions. All in all, this information should allow for the identification of more efficient measures to minimize both barrier effects and the risk of mortality.

## Results

**Figure 2:**
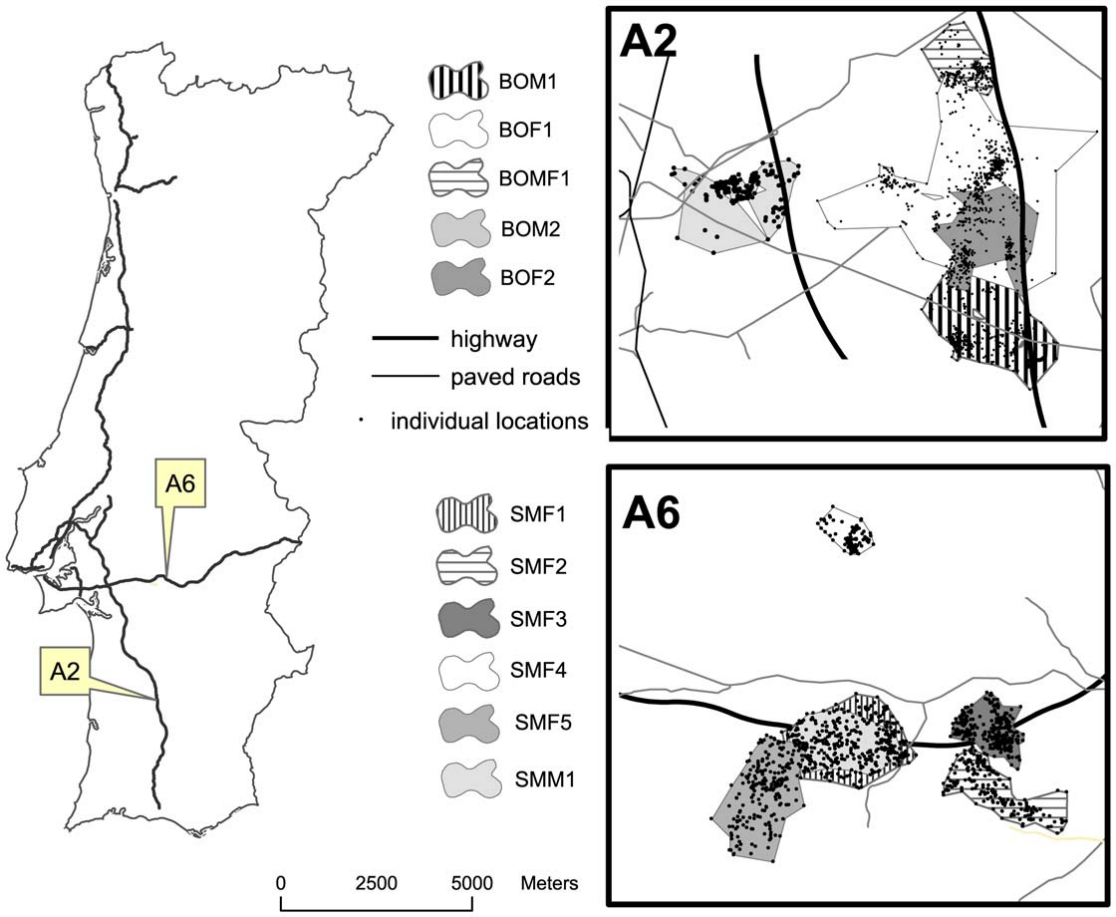
Highway A2 and A6 location in Portugal. Barn owls and stone martens NNCH home-ranges and locations for each individual (initials of species common name, sex and number).

We marked 11 adult barn owls near their nesting places; two individuals were at least one year old (BOM2 and BOF4), the remaining individuals were two years or older (Table 1). All stone martens were adults except SMF4 (Table 1). Among the barn owls radio-tracked, four were killed on the highway (36%) and another four disappeared from the study area. Two martens were confirmed road-kills (18%) and five disappeared (Table 1). We obtained sufficient data to calculate a stable home range for five barn owls and six stone martens (Table 1). Home-ranges averaged (±SD) 763±650 ha and 336±188 ha for barn owls and stone martens, respectively. Interestingly, three stone martens included the highway in the areas of their home range with a higher use probability while barn owls established their home-ranges mostly in the vicinity of the highway, including them in areas of their home ranges with a lower probability of use (Figure 2).

**Table 1:** Summary of individual data: ID, sex, radio-tracking period, N (number of locations), Fate (alive, road-killed or disappeared) and home-range size for barn owls and stone martens using MCP and 20-NNCH methods with 100%, 60% and 20% isopleths (HR1, HR2 and HR3, respectively).

ID	Sex	Radio-tracking period	N	Fate	MCP	HR1	HR2	HR3
***barn owls***								
BOF1^*^	♀	5-May-2008 to 7-Mar-2009	470	Alive	2571	1881	80	3
BOF2^*^	♀	7-June-2008 to 15-Oct-2009	237	Alive	628	468	101	12
BOM1^*^	♂	8-Aug-2008 to 26-Jan-2009	377	Road-killed	897	729	73	7
BOM2^*^	♂	2-Feb-2009 to 13-May-2009	299	Road-killed	632	513	38	2
BOMF1^*^	?	3-Sept-2009 to 16-Oct-2009	252	Alive	250	226	52	6
BOM3	♂	7-May-2008 to 6-Aug-2008	143	Road-killed	642			
BOM4	♂	9-May-2008 to 2-June-2008	39	Disapeared	131			
BOF3	♀	5-Aug-2008 to 2-Sept-2008	50	Disapeared	605			
BOM5	♂	17-Feb-2009 to 20-Mar-2009	22	Disapeared	311			
BOF4	♀	31-Mar-2009 to 2-May-2009	96	Disapeared	2685			
BOF5	♀	12-May-2008 to 29-5-2008	42	Road-killed	34			
***stone martens***								
SMF1^*^	♀	24-May-2008 to 14-Aug-2008	205	Alive	570	550	328	59
SMF2^*^	♀	14-Jun-2008 to 13-Jan-2009	213	Disapeared	418	301	164	46
SMF3^*^	♀	16-Jun-2008 to 21-Dec-2008	300	Disapeared	242	205	96	34
SMF4^*^	♀	28 Aug-2008 to 22-Dec-2008	104	Alive	105	105	28	9
SMF5^*^	♀	3-Apr-2009 to 17-Jul-2009	219	Alive	537	486	261	48
SMM1^*^	♂	25-Jan-2009 to 14-Mar-2009	238	Disapeared	391	372	254	82
SMM2	♂	22-May-2008 to 6-Jun-2008	8	Disapeared	472			
SMF6	♀	14-Jun-2008 to 21-Jun-2008	22	Disapeared	751			
SMF7	♀	29-Jul-2009 to 2-Sept-2009	189	Alive	489			
SMM3	♂	18-Sep-2009 to 21-Nov-2009	17	Road-killed	350			
SMM4	♂	13-Nov-2008 to 6-Dec-2008	62	Road-killed	263			

Individual's locations that reached asymptote of the curves accumulated area (^*^). Sex was undetermined for BOMF1.

### Habitat selection

We obtained a total of 2027 locations for barn owls and 1592 locations for stone martens. We were able to use 258 and 254 independent locations for each of the species. The presence of both species was negatively related to the distance to streams, while forest had a positive effect on the occurrence of stone martens (Table 2 and 3). Most importantly, the interaction term *D_highway*×*Traffic* is included in the most supported model for both species; indicating that individuals tended to avoid the highway when the traffic intensity increased (Table 2 and 3, Table S1).

**Table 2:** Summary of most supported models (ΔAIC≤2) for habitat selection, movements directionality within the home-range, movements directionality next to the highway, highway crossings and crossings *vs.* road-kill sites for barn owls and stone martens: total number of candidate models ( ), AIC (Akaike Information Criterion), ΔAIC (AIC_i_-_min_AIC), W_i_ (Akaike weight).

	AIC	ΔAIC	W_i_
***Habitat selection***			
*barn owl (14)*			
D_highway×Traffic+D_streams	662.0	0.0	0.68
*Null model*	719.3	57.3	
*stone marten (14)*			
D_highway×Traffic+D_streams+Forest	691.5	0.0	0.96
*Null model*	708.2	708.2	
***Movement directionality within the home- range***			
*barn owl (5)*			
D_highway*S_l_*	2005	0	0.99
*Null model*	2024	19	
*stone marten (5)*			
D_highway*S_l_*	937.4	0	1.00
*Null model*	1016	79	
***Movement directionality next to the highway***			
*barn owl (11)*			
D_highway*S_l_*+D_streams+Herbs	366.8	0.0	0.82
*Null model*	383.7	16.9	
*stone marten (11)*			
D_highway*S_l_*+D_streams+Treeshrub	416.8	0.0	0.83
*Null model*	437.7	20.9	
***Highway crossings***			
*barn owl (14)*			
Verge width+D_above-grade+Herbs	78.6	0.0	0.25
Verge width+D_above-grade+Herbs+Light vehicle traffic	79.38	0.8	0.19
Light vehicle traffic+D_croplands	79.65	1.1	0.17
Verge width+D_above-grade+Light vehicle traffic+D_croplands+Herbs	80.06	1.5	0.14
Verge width+D_above-grade	80.46	1.9	0.11
*Null model*	84.4	6.9	
*stone marten (15)*			
D_flat+Verge width	196.1	0	0.22
D_flat	196.9	0.8	0.15
D_flat+Verge width+Treeshrubs	197.8	1.7	0.10
D_flat+Verge width+Truck traffic	198	1.9	0.09
D_flat+Verge width+D_forest	197.9	1.8	0.09
*Null model*	198.1	2	
***Crossing vs. road-kill sites***			
*barn owl (11)*			
Traffic	46.45	0	0.51
*Null model*	49.05	2.6	
*stone marten (11)*			
Traffic+D_above-grade+D_allpassages	66.61	0	0.300
D_above-grade	67.48	0.9	0.194
Traffic+D_above-grade	67.57	1.0	0.186
*Null model*	73.71	6.6	

**Table 3:** Estimated coefficients (β), standard error (SE) and significance (p-value) for the most supported models for each species (ΔAIC≤2) (^*^ averaged model when we obtained more than one most supported model [60]).

Variables	β	SE	p-value
***Habitat selection***			
*barn owl*			
(intercept)	−0.423	0.211	0.045
D_streams	−0.0002	0.0001	0.140
D_highways×Traffic	0.000002	0.0000003	<0.001
*stone marten*			
(intercept)	−0.445	0.339	0.189
Forest	0.075	0.035	0.031
D_streams	−0.001	0.0005	0.002
D_highways×Traffic	0.000001	0.0000004	<0.001
***Movement directionality within the home-range***			
*barn owl*			
(intercept)	−0.361	0.107	<0.001
D_highway*S_l_*	0.0003	0.0001	<0.001
*stone marten*			
(intercept)	−1.197	0.120	<0.001
D_highway*S_l_*	0.001	0.001	<0.001
***Movement directionality next to the highway***			
*barn owl*			
(intercept)	−1.103	0.322	0.001
D_streams	−0.001	<0.001	0.018
Herbs	0.017	0.007	0.016
D_highway*S_l_*	0.002	0.001	0.026
*stone marten*			
(intercept)	−1.479	0.299	<0.001
D_streams	−0.002	0.001	0.012
Treeshrub	0.018	0.010	0.077
D_highway*S_l_*	0.005	0.001	<0.001
***Highway crossings***			
barn owl*			
(intercept)	−2.141	1.282	0.033
Verge width	0.264	0.095	0.005
D_above-grade	−0.002	0.004	0.079
Herbs	0.019	0.011	0.097
Light traffic	−0.001	0.001	0.240
D_open	−0.005	0.004	0.300
stone marten^*^			
(intercept)	1.346	0.084	0.189
D_flat	0.001	0.0004	0.024
Verge width	−0.322	0.196	0.102
Treeshrubs	−0.008	0.017	0.635
D_forest	−0.004	0.009	0.688
Truck traffic	−0.004	0.011	0.747
***Crossing vs. Road-kill sites***			
*barn owl*			
(intercept)	1.863	0.607	0.002
Traffic	−0.003	0.002	0.055
stone marten^*^			
(intercept)	7.290	1.956	<0.001
D_above-grade	−0.029	0.009	0.004
Traffic	−0.007	0.003	0.003
D_allpassages	−0.004	0.002	0.106

### Movement directionality within home ranges and next to the highway

We identified 721 and 421 movements towards the highway (TOW) and 736 and 416 movements away from the highway (AWA) for barn owls and stone martens, respectively. As expected, the distance from the starting point of the segment to the highway was the most relevant factor for both species (Table 2 and 3, Table S2). Both species avoided moving toward the highway when they were in close proximity to it (Table 3). Barn owls were 50% likely to move towards the highway at 1203 m from the highway, while this threshold for stone martens was at 950 m. Apparently, their movements show some avoidance to the highway but are also consistent with the radius of the home ranges, indicating that the overall directionality of individual movements within home ranges may not be affected by the highway. Traffic had no effect on the overall directionality.

When considering only those movements in the vicinity of the highway we found that landscape variables describing habitat quality were related to movement directionality. The most supported model for both species demonstrated that movements toward the highway were explained primarily by a high percentage of suitable vegetation in the road verges (herbaceous cover for barn owls and tree and shrubs for stone martens, Tables 2 and 3) and where the highway crossed streams. A distant starting point was also included in the model, but with a lower effect than the vegetation on the road verges (Table 2 and 3, Table S3).

### Highway crossings and road-kill sites

Six of the marked barn owls and five of the marked stone martens crossed the highway during our monitoring. Barn owls crossed the highway 29 times during 1175 hours of radio-tracking, while stone martens did so on 70 occasions during 866 hours of monitoring. Therefore, barn owls and stone martens living next to the highway crossed it approximately 0.30 and 0.97 times per day (assuming 12 hour activity period), respectively. Using data from all marked barn owls we calculated a risk of being road-killed per crossing event *r* of 0.009 and 0.018 (considering only those individuals actually killed and assuming all disappeared individuals as road-kills, respectively). Stone martens were less vulnerable than barn owls, with *r* values of 0.002 and 0.007, for the same respective groups.

Barn owl highway crossings were best explained by models containing verge features (width, topography, and vegetation). The likelihood of a barn owl crossing a highway was higher at sections that were above-grade, with wide road verges and a higher proportion of herbaceous cover in the verge (Table 3, Table S4). Stone martens tended to cross at narrow road verges and far from the leveled sections of the highway. Interestingly, barn owl highway crossings were associated with lower light-vehicle whereas stone marten crossings were related with truck traffic intensities (Table 2 and 3). As expected, high habitat suitability in close proximity to the highway also explained crossings for barn owls (croplands near the highway) and stone martens (tree and shrubs on the verges and forest next to the highway) (Table 3).

Between January 2003 and December 2009, BRISA recorded 11 barn owls and 13 stone martens killed within the section of highways covered by the home ranges of the individuals tracked. The comparison between the sites used for crossing and the sites where road-kills were found demonstrated that owls tended to be killed on highway sections where they preferred to cross but when traffic was high (Tables 2 and 3). Stone martens, on the other hand, tended to cross successfully on above-grade sections and close to existing passages and when traffic was low (Tables 2 and 3, Table S5). All the analysis performed showed low correlation for all distances and no departures from the model assumptions were detected.

## Discussion

A more integrated understanding of the relationship between road-kills and barrier effects can help provide for the more efficient management of roads and their environmental impacts. We explored this integration by investigating the individual behavioral responses towards roads at different spatial resolutions in two species commonly found as road casualties, providing valuable information on the factors associated with the risk of mortality [34]. We found that barn owls and stone martens showed different spatial responses towards highways at different spatial resolutions, and, more importantly, that the variables associated with those responses also change.

### Home-ranges and habitat selection

The first response of individuals towards highways occurs when establishing their home ranges. Our study shows that if there is available habitat, barn owls and stone martens may not avoid locating their home ranges in the vicinity of the highways, at least at the traffic volumes we observed. However, given the location of territories, highways are not barriers to movement but may be acting as an artificial home range boundary for barn owls. In fact, there are many examples of species using linear structures to define boundaries [12], [35]. Stone martens, on the other hand, were able to include the highway entirely within their home ranges, with individuals using areas located on both sides of the infrastructure, demonstrating that for some species the presence of highways and their associated traffic may not be the main determinant of home range use.

As expected, barn owls and stone martens used those areas with a higher habitat quality, including those in the vicinity of the highways. Interestingly, traffic intensity affected the habitat use of both species, but seems to have no influence on the decision to cross or move next to the highway. Stone martens and barn owls in particular relied greatly on their auditory system to locate prey - a critical factor in hunting [36]. Additionally, barn owls use vocal communications to mark their territories, and during adult-nestling, feeding interactions and therefore traffic noise can be an important source of disturbance [37]–[40].

### Staying or crossing

Both species avoided using the vicinity of the highway when traffic intensity was high. Nevertheless, individuals moved towards the highway when in close proximity to streams and in places where verges offered suitable habitat, i.e., locations at which they may encounter a high abundance of small mammals [41]. Stone martens regularly crossed the highway, particularly at narrow verges and non-leveled sections. Road sections at the same level as the surroundings may make individuals less aware of traffic, probably contributing to the higher probability of being road-killed. This is consistent with road-kill data that stone martens tend to be successful when crossing in above-grade sections [33]. This species also uses passage structures, and therefore it is very interesting to note that crossing events occurred irrespective of the presence of culverts or other structures [42], [43]. Nevertheless, successful crossings occurred near the existing passages. Thus, stone martens may use the passages but for passages to be used, they must be located at sites where individuals are willing to cross [44].

Barn owls, on the other hand, seem particularly vulnerable to be killed when crossing highways, even though they crossed less often than martens. Some highway sections may be functioning as attractive sinks, especially those with suitable verges (wide with herbaceous cover) and the ones at which barn owls tend to cross (above-grade sections). Barn owls frequently locate their prey by flying only 1–3 meters above the ground [45]. Above-grade sections do not prevent them from flying low, increasing the likelihood of being hit by a vehicle. Our analyses show that mortality risk increases when traffic intensity is higher; however, barn owls tended to cross when light-vehicle traffic was low (continuous but less noisy) whereas stone martens were sensitive only to truck traffic (discontinuous but noisy). The low traffic intensity during the hours in which both species cross the highway may increase crossing success.

### Mortality patterns

The crossing and mortality rates of the individuals monitored during our study allowed us to estimate the risk of being killed at each crossing event, which in turn can be translated into a potential mortality rate of 48–96 barn owls and 70–245 stone martens per 100 km of highway each year (assuming immediate replacement of dead individuals by immigrants and constant densities of 0.24 individuals/km^2^ for barn owls [46] and 0.76 individuals/km^2^ for stone martens [32]). These estimates are much higher than the mortality data collected by BRISA for the surveyed highway sections (6.2 ind./100 km/year and 26.6 ind./100 km/year, respectively for barn owls and stone martens). Even if crude road-kill counts always underestimate total mortality given that they do not take into account the detectability of victims and their rate of loss [47], we hypothesize that the main reason behind the disparity in estimates is that populations next to highways may act as sinks on a regional level. In other words, the actual density next to the road is probably much lower than it could be given the actual habitat quality. Breeding performance may to be affected by road density [48]. It may be that the high death toll imposed by highways may decrease density over time [49], [50] because there are not enough individuals available regionally to fill the gaps of the territories, following the death of the occupants. In fact, the much greater trapping effort that we needed to capture stone martens in the vicinity of the highway compared with that required in the same region further from roads (504 versus 109 trap-nights per capture, [32]) tends to support this interpretation. Finally, GRILO et al. [8] demonstrated that roads in well-connected habitats may act as sinks due to high stone marten-vehicle collisions. A similar explanation is attributed to breeding bird occurrence, where mortality is likely to be the cause of the negative relationship found between bird richness and abundance and distance to roads [51].

### Conclusions

Our study show that highways with this traffic intensity do not act as barrier to barn owl and stone marten movements. In contrast, we observed high mortality rates and found several road features that may increase the risk of mortality. Thus, mortality may be the primary road-mediated mechanism that may threatens barn owl and stone marten populations. Apart from abundance, road-related mortality can also change the demographic structure of populations [49], [52] and seems to have a much higher impact on genetic diversity than barrier effects [50]. Although barn owls and stone martens are not threatened, they provide valuable insight regarding individual spatial responses towards roads and how this behavior translates into a pattern of road-mortality. Land managers could reduce road mortality risk by decreasing sources of attraction, particularly for the more vulnerable species like the barn owl, and increasing road permeability through measures that promote safe crossings. Nevertheless, understanding which effective measures should be applied to minimize both negative effects is still needed. Thus, we recommend exploring the response of individuals to a reduction in prey alongside verges and raising the height of roadside verges in road sections with mortality. Reducing prey alongside those road sections could be done by changing the vegetation next to road-verges (e.g., ploughing). The importance of roadside verges as refuge habitats for small mammals in agricultural landscapes has recently been recognized [53]. Thus, complementary corridors of suitable grassland with the same verge width should be left beyond the road verge, parallel to the road. Raising the height of the roadside verges (similar to noise control earth berms [54]) may not only encourage owls to fly above traffic but also increase stone marten awareness of roads and traffic. Moreover, the importance of passages suggested by a previous study was not fully supported here with radio-tracking data. The simultaneous survey of passage use and movements through radio-tracking should clarify the role of existing passages and their characteristics for species to cross the highways safely, and will facilitate a more efficient deployment of corrective measures.

## Materials and Methods

This study was conducted along two highway sections under private concession by BRISA Auto- Estradas de Portugal, S.A. in Alentejo province, Southern Portugal: 69.5 km on the A2 for barn owls and 18.7 km on the A6 for stone martens (Figure 2). Both highways had four-lanes, a 7 m wide median strip, livestock exclusion fencing on both sides and a speed limit of 120 km/h. The A2 section was built between 1997 and 2001. Its annual average daily traffic intensity (AADT) is 13949 vehicles/day (5536 vehicles/day between 6pm and 6am). This section of highway cuts through plains dominated by open extensive croplands, which a priori represents suitable habitat for barn owls [55], as shown by the incidence of barn owl road mortality (14 road-killed barn owls/100 km/year, BRISA unpublished data). The A6 section was built in 1995, with current AADT volumes of 8373 vehicles/day (3011 vehicles/day between 6pm and 6am). This section of highway runs through an area with elevations ranging from 200 m to 500 m a.s.l that is dominated by savannah-like woods of cork oak *Quercus suber* and holm oak *Q. ilex* (hereafter forest), representing suitable habitat for the stone marten in Mediterranean regions of the Iberian peninsula [32]. The section includes a high incidence of stone marten mortality with a registered kill rate of 23 stone martens/100 km/year.

### Field protocols

Between April 2008 and September 2009 we marked and radio-tracked 11 barn owls and 11 stone martens. All individuals were captured at locations less than 1.5 km from the highways (average home-range radius of both the barn owl and stone marten, [46], [32] respectively), except for one stone marten (captured 4 km from the highway). We captured barn owls by using hoop nets or mist nets inside or in the vicinity of abandoned houses [56]. Stone martens were captured with box-traps (Tomahawk Live Trap Co., Wisconsin, USA) baited with carrion (sardine, chicken wings). Both species were radio-tagged with VHF radio-transmitters (Biotrack LTD TW-3 single celled tag and Telonics 80 with activity sensor, for barn owls and stone martens, respectively). Stone martens were anesthetized to be handled (Imalgene 1000 and Midazolan). Individuals were examined for general body condition, measured, weighed and released at their capture site. The capture and handling of both species was conducted under the required legal permits (ICNB/CEMPA Licenses 105/2008/CAPT; 39/2009/CAPT; 40/2009/CAPT; 168/2009/CAPT). Since the probability of being killed on the road was expected to be high, we monitored each individual from dusk to dawn, obtaining locations with successive triangulations every 30 min. The bearings were taken synchronously by two observers, from independent points, using hand-held three-element Yagi antennas. When individuals approached the highway, one of the bearings was obtained from the nearest overpass. We estimated our location error using hidden radio-transmitters at different known heights and positions within the study area. The estimated error was 196±124 m (mean ± SD, n = 35) and 182±155 m (n = 106), respectively for barn owl transmitters and stone marten collars. Additionally, BRISA Auto-estradas de Portugal S.A. provided barn owl and stone marten road-kill data for the highway sections between 2003 and 2009.

### Home-range location and size

We used the Local Nearest Neighbor Convex-Hull (NNCH) method to estimate the location and size of home-ranges using the LoCoH Home-range Generator for ArcGis 9.1 (ESRI, Redlands, CA, USA). The NNCH is an extension of the Minimum Convex Polygon (MCP) that identifies areas of high and low use density by taking the union of the MCP associated with k-1 nearest neighbors [57]. We used k = 20 neighbors to estimate home-ranges and the quantile algorithm to calculate the isopleths (100, 60 and 20). We estimated MCP home-range areas for all individuals, distinguishing between those with and without a sample size sufficiently large to obtain a stable home-range size. By definition individuals with stable home-ranges are expected to reach an asymptote while floaters and under-sampled individuals increase their ranges progressively. The threshold sample size using incremental area plots was estimated by adding successive locations for all tracked individuals.

### Habitat selection

We evaluated the effect of road- and landscape-related features on the individual's locations by comparing the properties at the actual radio locations with the data obtained from a set of random points obtained within each home-range. We used the Schoener's Index (>1.6 and <2.4) to select independent locations [58]. For each home-range we estimated the same number of random points as locations. Each location/random point was buffered with a radius corresponding to the mean location error and described in terms of road- and landscape-related factors within this area (Table 4).

**Table 4:** Summary of road- and landscape-related features measured at locations points and highway crossings: scale, range for barn owls (*) and stone marten (**)).

Symbol	Variable description	Scale	Range	
Individual locations			barn owl	stone marten
*Road features*				
D_highway	Average distance to highway	m	68–4754	54–5783
D_paved	Average distance to two-lane paved roads	m	72–3505	52–4498
D_unpaved	Average distance to unpaved roads	m	19–803	26–738
Trafficψ	Number of vehicles that used the highway at the hour the exact location was taken	Vehicle/hour	0–2066	0–1055
*Landscape features*				
Croplands^*^	Croplands areas (pastures, extensive agriculture)	ha	0–12	-
Forest^**^	Forest area (cork oak woodlands)	ha	-	0–10
D_streams	Average distance from the main streams	m	44–2565	41–1348
D_urban	Average distance from urban areas	m	2645–9473	797–6465
D_buildings	Average distance from buildings	m	83–2460	55–1395
**Highway crossings**				
*Traffic*				
Traffic	Total traffic	Vehicle/hour	25–1469	23–1015
Light vehicle trafficψψ	Traffic class 1 and 2 vehicles	Vehicle/hour	10–1352	15–1002
Truck trafficψψ	Traffic class 3, 4 and 5 vehicles	Vehicle/hour	0–48	0–44
*Verges*				
Verge width	Average distance from highway to soil	m	2–20	5–9
D_below-grade	Average distance to below-grade or entrenched highway sections (slope <−20 radians from soil to highway)	m	90–834	92–289
D_flat	Average distance of same level of highway and soil (slope between −20 and +20 radians from soil to highway)	m	106–20058	102–1270
D_above-grade	Average distance to above-grade highway sections (slope >+20 radians from soil to highway)	m	90–1284	91–244
Herbs^* ^ ψψ	Average % of herbaceous cover	100 m	15–99	-
Treeshrubs^** ^ ψψ	Average % of trees and shrubs	100 m		26–76
*Habitat connectivity*				
D_croplands^* ^ ψψ	Average distance from croplands	m	0–323	-
D_forest^** ^ ψψ	Average distance from forest	m		0–112
D_allpassages	Average distance to the nearest culvert/underpass/overpass	m	-	20–5826

ψ was included as interaction variable with highway distance,

ψψ variable included in the movement directionality analysis.

Road-related features included average distance to the highway as well as to paved and unpaved roads. Traffic intensity was estimated as the number of vehicles that used the highway during the hour of the location. Because the influence of traffic is expected to decline as the distance to the road increases, we included an interaction term between highway traffic intensity and the distance to the highway, both obtained within the same hour (*D_highway×Traffic*; Table 2 and 3). In the case of random points we randomly assigned the values of traffic intensity observed from deleted dependent locations. Landscape-related variables included the distance to the nearest stream, urban area and building, and also the area of suitable habitat for each species (Table 4).

We used Generalized Linear Mixed Models (GLMM) with a binomial distribution and a logit link using independent locations/random points as the response variable [59] and individuals as a random effect to avoid pseudo-replication among individuals. We designed 14 candidate models for barn owls and stone martens using three sets of models assuming that: 1) habitat selection is mostly affected by the road and its disturbance, 2) landscape features are the most relevant variables to explain their spatial behavior, and 3) both road- and landscape-related features affect space use (best models obtained from 1) and 2)). We used an information-theoretic approach for model selection. Models were ranked according to Akaike's Information Criterion (AIC) [60]. We started with no variables in the model (null model) and sequentially entering variables one at a time according to the lowest AIC. To avoid multicollinearity, we did not enter into the same model variables with a Pearson correlation coefficient >0.5 (we dropped the one with a lower correlation with the dependent variable). We used a spline correlogram to investigate auto-correlation in the locations/random points (data not shown). Model accuracy was examined to assess how well the most parsimonious model fits the data using quantile-quantile plots (data not shown).

### Movement directionality within home ranges and next to highway

We monitored movements to assess whether, and if so how, these animals responded to the highway and its traffic. With these analyses we attempted to: 1) identify at which distance from the highway and at which traffic volume individuals were more likely to move toward the highway within their home-ranges, and 2) evaluate which road- and landscape-related features influenced movement directionality next to the highway. Thus, for the first objective we defined movements as sections joining two successive locations (time interval of 30±15 minutes) and then classified sections in two types: move towards the highway (TOW) and move away from the highway (AWA). We extracted the following variables: 1) distance from starting point of the segment to the highway, 2) light-vehicles intensity and 3) truck traffic intensity. We designed five candidate models using all combinations of non-correlated variables. We ran binomial GLMM models with a logit link using the type of movement as the response variable (TOW - 1; AWA - 0) and individuals as a random effect, as described in the habitat selection analysis.

To examine which road- and landscape-related features influenced the directionality of the movements next to the highway, we selected all movement sections within twice the mean location error distance to the highway for each species. Then, we extracted information on the road- and landscape-related features within a buffer with a radius equal to the mean location error of the starting location (*S_l_*) and final location (*F_l_*) of the segment. We used the distance to the highway at the starting point (*D_highwayS_l_*) and the difference of each variable by subtracting the value of the variable at *S_l_* from *F_l_* for the following road variables: light-vehicles and truck traffic, and the percentage of suitable road verges (trees and shrubs for stone martens and herbaceous cover for barn owls). A similar procedure was performed for forest/cropland area and distance to streams (landscape variables).

Candidate models were designed assuming three hypotheses regarding the movement of individuals toward the highway: 1) road-related features fully explain their movements, 2) movements are affected only by landscape-related features, and 3) both types of variables explain their movements. We ran binomial GLMM models with a logit link using the type of movement (TOW - 1; AWA - 0) as the response variable and individuals as a random effect, following the same procedures as described above.

### Highway crossings and road-kill sites

We used GLMM to describe highway crossings by comparing the crossing sites with random sites on the highway. Crossing sites were defined as the point where the line delineated by two consecutive locations (less than one hour apart) crossed the highway. We then described three groups of road-related variables within a buffer defined by the telemetry error (Table 4). Candidate models addressed three hypotheses: 1) traffic intensity is the main factor explaining the crossings, 2) crossings are explained by the verge features, and 3) crossings are defined by the local habitat connectivity expressed as the distance to suitable habitat (croplands/forest) and the presence of culverts/underpasses/overpasses for stone martens. Using similar procedures, we used logistic models to explore differences between the sites selected for crossing and the sites where some individuals were found road-killed.

Road-related variables were provided by BRISA (highways, culverts/underpasses/overpasses, traffic intensity) and IgeoE (two-lane paved roads). Fieldwork was performed to describe road verges every 100 m (type of vegetation, slope, verge width). A land use map was prepared using Google Earth (30 October 2006 image 2010 IGP/DGRF Europe Tecnologies, Tele Atlas) to identify forest, main streams, buildings, urban areas and unpaved roads. We calculated home-ranges using Arcview 3.2 and ArcGis 9.3 and the Animal Movement extension program [61]. We performed statistical tests using lme4 [62], glmmML [63], and ncf [64] packages in R version 2.13.2 [65].

## Supporting Information

Table S1 **Summary of the candidate habitat selection models for barn owl and stone marten: AIC (Akaike Information Criterion), ΔAIC (AIC_i_ -_min_AIC), W_i_ (Akaike weight).**
(DOCX)

Table S2 **Summary of the candidate models on barn owl and stone marten movements directionality within home-range: AIC (Akaike Information Criterion), ΔAIC (AIC_i_ -_min_AIC), W_i_ (Akaike weight).**
(DOCX)

Table S3 **Summary of the candidate movements directionality next to the highway models for barn owl and stone marten: AIC (Akaike Information Criterion), ΔAIC (AIC_i_ -_min_AIC), W_i_ (Akaike weight).**
(DOCX)

Table S4 **Summary of the candidate highway crossing models for barn owl and stone marten: AIC (Akaike Information Criterion), ΔAIC (AIC_i_ -_min_AIC), W_i_ (Akaike weight).**
(DOCX)

Table S5 **Summary of the candidate crossing** ***vs*** **. road-kill sites models for barn owl and stone marten: AIC (Akaike Information Criterion), ΔAIC (AIC_i_ -_min_AIC), W_i_ (Akaike weight).**
(DOCX)
