# Supplementary material for: Individual Spatial Responses towards Roads: Implications for Mortality Risk
Source: PLoS One. 2012 Sep 6;7(9):e43811. doi: 10.1371/journal.pone.0043811 (PMC3435373; doi:10.1371/journal.pone.0043811)
Supplement: Table S1 — Summary of the candidate habitat selection models for barn owl and stone marten: AIC (Akaike Information Criterion), ΔAIC (AICi -minAIC), Wi (Akaike weight). (DOCX) [file pone.0043811.s001.docx]

| **barn owl** | **AIC** | **ΔAIC** | **W_i_** |
| --- | --- | --- | --- |
| ***Road features*** |  |  |  |
| D_highway | 726.2 | 64.2 | <0.001 |
| D_paved | 726.5 | 64.5 | <0.001 |
| D_unpaved | 726.6 | 64.6 | <0.001 |
| D_highway x Traffic | 664.4 | 2.4 | 0.203 |
| D_highway x Traffic + D_paved | 666.1 | 4.1 | 0.087 |
| D_highway x Traffic + D_paved+ D_unpaved | 668 | 6 | 0.034 |
| ***Landscape features*** |  |  |  |
| Croplands | 720.8 | 58.8 | <0.001 |
| D_streams | 717.2 | 55.2 | <0.001 |
| D_urban | 725.4 | 63.4 | <0.001 |
| D_buildings | 725.9 | 63.9 | <0.001 |
| Croplands + D_streams | 719 | 57 | <0.001 |
| Croplands + D_streams + D_urban | 720 | 58 | <0.001 |
| Croplands + D_streams + D_urban + D_buildings | 722 | 60 | <0.001 |
| ***Road + landscape features*** |  |  |  |
| D_highway x Traffic + D_streams | 662 | 0 | 0.676 |
| *Null model* | 719.3 | 57.3 | <0.001 |
|  |  |  |  |
| **stone marten** |  |  |  |
| ***Road features*** |  |  |  |
| D_highway | 714.1 | 22.6 | <0.001 |
| D_paved | 715.4 | 23.9 | <0.001 |
| D_unpaved | 709.9 | 18.4 | <0.001 |
| D_highway x Traffic | 703.5 | 12.0 | 0.002 |
| D_highway x Traffic + D_unpaved | 705.2 | 13.7 | 0.001 |
| D_highway x Traffic + D_unpaved+ D_paved | 706.4 | 14.9 | <0.001 |
| ***Landscape features*** |  |  |  |
| Forest | 706 | 14.5 | <0.001 |
| D_streams | 701.2 | 9.7 | 0.007 |
| D_urban | 715.6 | 24.1 | <0.001 |
| D_buildings | 715.4 | 23.9 | <0.001 |
| Forest + D_streams | 699.3 | 7.8 | 0.020 |
| Forest + D_streams + D_buildings | 701.3 | 9.8 | 0.007 |
| Forest + D_streams + D_buildings + D_urban | 706.9 | 15.4 | <0.001 |
| ***Road + landscape features*** |  |  |  |
| D_highway x Traffic + Forest + D_streams | 691.5 | 0.00 | 0.956 |
| *Null model* | 708.2 | 708.20 | <0.001 |
