# Supplementary material for: Individual Spatial Responses towards Roads: Implications for Mortality Risk
Source: PLoS One. 2012 Sep 6;7(9):e43811. doi: 10.1371/journal.pone.0043811 (PMC3435373; doi:10.1371/journal.pone.0043811)
Supplement: Table S2 — Summary of the candidate models on barn owl and stone marten movements directionality within home-range: AIC (Akaike Information Criterion), ΔAIC (AICi -minAIC), Wi (Akaike weight). (DOCX) [file pone.0043811.s002.docx]

| **barn owls** | **AIC** | **ΔAIC** | **W_i_** |
| --- | --- | --- | --- |
| D_highways*S_l_* | 2005 | 0 | 0.998 |
| Light vehicle traffic | 2028 | 23 | <0.001 |
| Truck traffic | 2025 | 20 | <0.001 |
| D_highways*S_l_* x Light vehicle traffic | 2026 | 21 | <0.001 |
| D_highways*S_l_* x Truck traffic | 2018 | 13 | 0.002 |
| *Null model* | 2024 | 19 | <0.001 |
|  |  |  |  |
| **stone marten** |  |  |  |
| D_highways*S_l_* | 937.4 | 0 | 1.000 |
| Light vehicle traffic | 1118 | 181 | <0.001 |
| Truck traffic | 1118 | 181 | <0.001 |
| D_highways*S_l_* x Light vehicle traffic | 1097 | 160 | <0.001 |
| D_highways*S_l_* x Truck traffic | 1031 | 94 | <0.001 |
| *Null model* | 1016 | 79 | <0.001 |
