# Supplementary material for: Individual Spatial Responses towards Roads: Implications for Mortality Risk
Source: PLoS One. 2012 Sep 6;7(9):e43811. doi: 10.1371/journal.pone.0043811 (PMC3435373; doi:10.1371/journal.pone.0043811)
Supplement: Table S3 — Summary of the candidate movements directionality next to the highway models for barn owl and stone marten: AIC (Akaike Information Criterion), ΔAIC (AICi -minAIC), Wi (Akaike weight). (DOCX) [file pone.0043811.s003.docx]

| **barn owls** | **AIC** | **ΔAIC** | **W_i_** |
| --- | --- | --- | --- |
| ***Road features*** |  |  |  |
| D_highways*S_l_* | 377.3 | 10.5 | 0.004 |
| Light vehicle traffic | 386.1 | 19.3 | <0.001 |
| Truck traffic | 385.1 | 18.3 | <0.001 |
| Herbs | 373.9 | 7.1 | 0.023 |
| Herbs + D_highways*S_l_* | 370.9 | 4.1 | 0.105 |
| Herbs + D_highways*S_l_* + Truck traffic | 373.9 | 7.1 | 0.023 |
| Herbs + D_highways*S_l_* + Truck traffic + Light vehicle traffic | 373.6 | 6.8 | 0.027 |
| ***Landscape features*** |  |  |  |
| Croplands | 384.9 | 18.1 | <0.001 |
| D_streams | 380.0 | 13.2 | 0.001 |
| D_streams + Croplands | 381.5 | 14.7 | 0.001 |
| ***Road + landscape features*** |  |  |  |
| Herbs + D_highways*S_l_* + D_streams | 366.8 | 0.0 | 0.815 |
| *Null model* | 383.7 | 16.9 |  |
|  |  |  |  |
| **stone marten** |  |  |  |
| ***Road features*** |  |  |  |
| D_highways*S_l_* | 422.9 | 6.1 | 0.039 |
| Light vehicle traffic | 439.7 | 22.9 | <0.001 |
| Truck traffic | 438.7 | 21.9 | <0.001 |
| Treeshrub | 433.8 | 17.0 | <0.001 |
| D_highways*S_l_* + Treeshrub | 421.6 | 4.8 | 0.075 |
| D_highways*S_l_* + Treeshrub+ Truck traffic | 422.9 | 6.1 | 0.039 |
| D_highways*S_l_* + Treeshrub+ Truck traffic+ Light vehicle traffic | 424.9 | 8.1 | 0.014 |
| ***Landscape features*** |  |  |  |
| Forest | 438.9 | 22.1 | <0.001 |
| D_streams | 433.6 | 16.8 | <0.001 |
| D_streams + Forest | 435.2 | 18.4 | <0.001 |
| ***Road + landscape features*** |  |  |  |
| D_highway*S_l_* + Treeshrub + D_streams | 416.8 | 0.0 | 0.831 |
| *Null model* | 437.7 | 20.9 |  |
