# Supplementary material for: Individual Spatial Responses towards Roads: Implications for Mortality Risk
Source: PLoS One. 2012 Sep 6;7(9):e43811. doi: 10.1371/journal.pone.0043811 (PMC3435373; doi:10.1371/journal.pone.0043811)
Supplement: Table S4 — Summary of the candidate highway crossing models for barn owl and stone marten: AIC (Akaike Information Criterion), ΔAIC (AICi -minAIC), Wi (Akaike weight). (DOCX) [file pone.0043811.s004.docx]

| **barn owls** | **AIC** | **Δ AIC** | **W_i_** |
| --- | --- | --- | --- |
| ***Traffic*** |  |  |  |
| Light vehicle traffic | 84.87 | 6.3 | 0.012 |
| Truck traffic | 86.36 | 7.8 | 0.006 |
| Light vehicle traffic + Truck traffic | 86.86 | 8.3 | 0.005 |
| ***Verges*** |  |  |  |
| Verge width | 83.17 | 4.6 | 0.029 |
| D_above-grade | 84.83 | 6.2 | 0.013 |
| Herbs | 85.1 | 6.5 | 0.011 |
| Verge width + D_above-grade | 80.46 | 1.9 | 0.112 |
| Verge width + D_above-grade+Herbs | 78.6 | 0.0 | 0.258 |
| ***Habitat connectivity*** |  |  |  |
| D_open | 85.86 | 7.3 | 0.008 |
| ***Traffic + Verges*** |  |  |  |
| Light vehicle traffic +Verge width + D_above-grade + Herbs | 79.38 | 0.8 | 0.193 |
| ***Traffic + Habitat connectivity*** |  |  |  |
| Light vehicle traffic + D_croplands | 79.65 | 1.1 | 0.168 |
| ***Verges + Habitat connectivity*** |  |  |  |
| Verge width+D_above-grade+Herbs+ D_croplands | 86.28 | 7.7 | 0.006 |
| ***Traffic + Verges+Habitat connectivity*** |  |  |  |
| Light vehicle traffic + Verge width + D_above-grade + Herbs + D_croplands | 80.06 | 1.5 | 0.137 |
| *Null model* | 84.4 | 5.8 |  |
|  |  |  |  |
| **stone marten** |  |  |  |
| ***Traffic*** |  |  |  |
| Light vehicle traffic | 202.3 | 6.2 | 0.010 |
| Truck traffic | 200.1 | 4 | 0.030 |
| Light vehicle traffic + Truck traffic | 204.3 | 8.2 | 0.004 |
| ***Verges*** |  |  |  |
| Verge width | 199.8 | 3.7 | 0.035 |
| D_flat | 196.9 | 0.8 | 0.151 |
| Treeshrubs | 200 | 3.9 | 0.032 |
| D_flat + Verge width | 196.1 | 0 | 0.225 |
| D_flat + Verge width + Treeshrubs | 197.8 | 1.7 | 0.096 |
| ***Habitat connectivity*** |  |  |  |
| D_forest | 199.2 | 3.1 | 0.048 |
| D_allpassages | 199.9 | 3.8 | 0.034 |
| D_forest + D_allpassages | 201 | 4.9 | 0.019 |
| ***Traffic + Verges*** |  |  |  |
| Truck traffic + D_flat + Verge width | 198 | 1.9 | 0.087 |
| ***Traffic + Habitat connectivity*** |  |  |  |
| Truck traffic + D_forest | 201.2 | 5.1 | 0.018 |
| ***Verges + Habitat connectivity*** |  |  |  |
| D_flat + Verge width + D_forest | 197.9 | 1.8 | 0.092 |
| ***Traffic + Verges + Habitat connectivity*** |  |  |  |
| Truck traffic + D_flat + Verge width + D_forest | 199.8 | 3.7 | 0.035 |
| *Null model* | 198.1 | 2 |  |
