# Supplementary material for: Individual Spatial Responses towards Roads: Implications for Mortality Risk
Source: PLoS One. 2012 Sep 6;7(9):e43811. doi: 10.1371/journal.pone.0043811 (PMC3435373; doi:10.1371/journal.pone.0043811)
Supplement: Table S5 — Summary of the candidate crossing vs . road-kill sites models for barn owl and stone marten: AIC (Akaike Information Criterion), ΔAIC (AICi -minAIC), Wi (Akaike weight). (DOCX) [file pone.0043811.s005.docx]

| **barn owls** | **AIC** | **Δ AIC** | **W_i_** |
| --- | --- | --- | --- |
| ***Traffic*** |  |  |  |
| Traffic | 46.45 | 0.0 | 0.427 |
| ***Verges*** |  |  |  |
| D_below | 50.89 | 4.5 | 0.046 |
| D_flat | 50.44 | 4.0 | 0.058 |
| Verge width | 50.85 | 4.4 | 0.047 |
| Herbs | 50.95 | 4.6 | 0.045 |
| ***Habitat connectivity*** |  |  |  |
| D_croplands | 50.62 | 4.2 | 0.053 |
| ***Traffic + Verges*** |  |  |  |
| Traffic + D_flat | 48.40 | 2.1 | 0.169 |
| ***Traffic + Habitat connectivity*** |  |  |  |
| Traffic + D_croplands | 49.38 | 2.9 | 0.099 |
| ***Verges + Habitat connectivity*** |  |  |  |
| D_flat + D_croplands | 52.40 | 6.0 | 0.022 |
| ***Traffic + Verges + Habitat connectivity*** |  |  |  |
| Traffic + D_flat + D_croplands | 50.3 | 3.8 | 0.063 |
| *Null model* | 49.05 | 2.7 |  |
|  |  |  |  |
| **stone marten** |  |  |  |
| ***Traffic*** |  |  |  |
| Traffic | 74.78 | 8.2 | 0.005 |
| ***Verges*** |  |  |  |
| D_flat | 70.27 | 3.7 | 0.048 |
| D_above-grade | 67.48 | 0.9 | 0.194 |
| Verge width | 75.66 | 9.1 | 0.003 |
| Treeshrubs | 73.41 | 6.8 | 0.010 |
| ***Habitat connectivity*** |  |  |  |
| D_forest | 75.02 | 8.4 | 0.004 |
| D_allpassages | 74.31 | 7.7 | 0.006 |
| ***Traffic + Verges*** |  |  |  |
| Traffic + D_above-grade | 67.57 | 1.0 | 0.186 |
| ***Traffic + Habitat connectivity*** |  |  |  |
| Traffic + D_allpassages | 75.19 | 8.6 | 0.004 |
| ***Traffic + Verges + Habitat connectivity*** |  |  |  |
| Traffic + D_above-grade + D_allpassages | 66.61 | 0.0 | 0.300 |
| *Null model* | 73.7 | 6.2 | 0.016 |
